# The Role of Exosomes as a Key Factor of Cytostatic Resistance in Cancer: Mechanisms of Action, Potential Biomarkers, and Possible Exosome-Based Therapies

**DOI:** 10.32604/or.2025.070356

**Published:** 2025-12-30

**Authors:** Sandra Kałużna, Monika Świerczewska, Sylwia Ciesiółka, Małgorzata Partyka, Michał Nowicki, Karolina Wojtowicz

**Affiliations:** 1 Department of Histology and Embryology, University of Medical Sciences in Poznan, Święcickiego 6 St. , Poznań, 60-781, Poland; 2 Institute of Health Sciences, Collegium Medicum, University of Zielona Góra, Zyty 28 St., Zielona Góra, 65-046, Poland

**Keywords:** Exosomes, drug resistance, cancer

## Abstract

The last research focuses on the role of exosomes in cancer treatment. Exosomes are extracellular vesicles. They can be secreted by cancer cells, and they can modulate chemotherapy sensitivity. Determining exosomal content opens the possibility for guiding treatment strategies for cancer diseases. Exosomal microRNA are considered one of the prime candidates for exosomal biomarkers. Exosomal circular RNAs represent excellent biomarkers for liquid biopsy because of their stability in many types of cancer. Exosomal proteins remain reliable biomarkers also. Exosomes have emerged as promising therapeutic candidates. Their biological properties render them ideal vectors for drug delivery. Genetic modification of exosomes is an effective way to deliver material capable of modulating cellular pathways involved in drug resistance. Furthermore, exosomes have been explored as carriers for metal-chelating agents. Integrating exosome-based therapies with traditional anticancer agents aims to exploit the natural targeting abilities of exosomes to enhance drug delivery. Despite the dynamic development of this field, many mechanisms of exosome action remain incompletely understood. Therefore, it is necessary to conduct further studies that will allow for a better understanding of their role in the process of resistance and will enable the development of effective therapeutic strategies.

## 1 Introduction

The incidence of cancer worldwide is increasing annually. Cancer is one of the most deadly diseases, causing about 20% of all deaths in Europe [1]. Cancer therapy is tailored to the individual patient based on the type and stage of the cancer. Treatment may include surgery, radiotherapy, chemotherapy, immunotherapy, or targeted therapy [2]. Chemotherapy involving cytotoxic drugs is the most commonly chosen treatment method. Cytostatics are toxic to cancer cells because they cause damage to the DNA of these cells or disrupt the processes necessary for their division. The result is inhibition of tumor development or its reduction [3].

A significant obstacle to chemotherapy’s success is drug resistance development. This resistance can be innate, present before treatment, or acquired, developing during treatment. The complex interaction between these factors contributes to developing resistance to various anticancer therapies. Intrinsic factors include pre-existing genetic mutations, tumor heterogeneity, and activated intracellular defence pathways. This leads to, among other things, the activation of intracellular defence pathways, which enable drug evasion by modulating oncogenic pathways, altering therapeutic targets, enhancing DNA repair, and activating survival mechanisms. In turn, extrinsic factors, primarily components of the tumor microenvironment (TME), actively contribute to developing cancer cells’ ability to evade the cytotoxic effects of anticancer drugs [4]. It is well established that multiple factors modulate the activity of recipient cells. Exosomes carry a variety of molecules derived mainly from cancer cells, epithelial and mesenchymal cells, and immune cells such as B lymphocytes and dendritic cells. Through this cargo, exosomes influence recipient cells, affecting their proliferation and functional activity in innate and adaptive immune systems. For instance, CD4+ and CD8+ T cells, including cytotoxic T lymphocytes (CTLs), can be directly or indirectly stimulated or suppressed in their proliferation and functions by exosome-mediated signaling [5].

Drug resistance is the primary and natural ability of cells to protect themselves from the toxic effects of drugs. It can also appear during the treatment of oncology patients as secondary drug resistance. Thus, cytostatic resistance is a severe challenge in the treatment of cancer. Cancer cells respond less to treatment or become completely resistant when it occurs. Acquired resistance significantly reduces the effectiveness of chemotherapy administered by physicians, and in many cases, contributes to the recurrence of cancer [6]. Up to 90% of cancer-related deaths are attributed to ineffective pharmacological treatment due to drug resistance [6,7]. Cancer cell plasticity may be key in this process. It has been shown that cancer cells can adapt to changing conditions to maintain their resistance, which results in a dynamically decreasing effectiveness of therapy [8].

Exosomes act as resistance vectors in many cancers, transferring resistant traits to treatment-sensitive cells. Understanding the mechanisms by which exosomes influence resistance is significant, as it may lead to the development of effective anticancer therapies. In breast cancer (BC), the second leading cause of cancer deaths among women, exosomal miR-9 can induce properties characteristic of cancer-associated fibroblasts (CAFs) [9]. In colorectal cancer, exosomal miR-1246 promotes mutations in tumor cells, induces M2 macrophage polarization, and remodels the tumor microenvironment by upregulating IL-10, transforming growth factor beta (TGFβ), and matrix metalloproteinase (MMP) expression [10]. In hepatocellular carcinoma (HCC), exosomal microRNAs (miRNAs) have also been shown to modulate tumor behavior and remodel the tumor microenvironment (TME) by regulating processes such as cell proliferation, metastasis, and immune response. [11]. In lung cancer, exosomes have been shown to modulate the tumor microenvironment by inducing phenotypic changes that hinder drug response. An example is TGF-β transported via exosomes, allowing cancer cells to transmit dormancy-inducing signals to neighboring cells, promoting the acquisition of a dormant phenotype. Consequently, dormant cells often exhibit reduced sensitivity to chemotherapy and targeted therapies, contributing to drug resistance [12].

The development of drug resistance in cancer cells is a highly complex process. Changes can occur at the molecular, cellular, and systemic levels. Contributing factors include: alterations in drug transport, changes in their metabolism or the target sites of action, dysregulation of apoptosis, modifications in DNA repair processes or the cell cycle. In addition, the phenotypic variability of the tumor and its microenvironment can have an impact.

Numerous studies have been conducted to better understand the resistance mechanisms to cytostatic drugs. It has been established that there are two main variants: congenital and acquired. They differ because the congenital variant exists before treatment, whereas the acquired variant develops in response to therapy, making it a greater challenge for physicians and researchers [6]. Signaling pathways that regulate fundamental biological processes, such as cell growth, proliferation, motility, angiogenesis, and survival, play a key role in drug resistance. Excessive activation of the phosphoinositide 3-kinase (PI3K) pathway is one of the most common cancer-related events in humans and contributes to tumor progression [13]. Conversely, the Gas6-Axl axis promotes cancer cell invasion by inducing multiple actin-dependent processes affecting cell dynamics, adhesion, and metabolism [14]. Disruptions in apoptotic pathways pose a significant challenge in cancer treatment. The primary role here is played by genes involved in regulating apoptosis, including p53 and members of the B-cell leukemia/lymphoma-2 (Bcl-2) family, which exhibit pro-survival activity in various cancers. It has been proven that a mutation in the TP53 gene encoding the transcription factor p53 inhibits the induction of apoptosis. As a result, cancers with TP53 mutations respond poorly to cytostatic therapy [15]. However, overexpression of Bcl-2 in cancer cells inhibits cell death pathways, resulting in lower effectiveness of anticancer drugs [16,17].

One of the most significant challenges in cancer therapy is multidrug resistance (MDR), characterised by cross-resistance to multiple drugs [6]. The most essential proteins contributing to MDR are drug transporters from the MDR protein family, including P-glycoprotein (P-gp) and breast cancer resistance protein (BCRP), which play a pivotal role [18–20]. The proteins involved in this process have been studied for many years. There is extensive literature on this subject, and our article does not focus strictly on this particular issue.

The role of the TME in shaping resistance to cytostatic drugs should not be underestimated, as its composition differs significantly from physiological conditions. Among the key components of the TME are the extracellular matrix (ECM), cytokines, chemokines, growth factors, and metabolic elements. ECM proteins play a crucial role in tumor progression and the development of treatment resistance. During neoplastic transformation, these proteins undergo functional changes that promote tumor cell survival and support their aggressive phenotype. The most prominent components of the ECM include collagens, elastin, laminins, fibronectin, lysyl oxidase (LOX), and transforming growth factor-beta-induced protein (TGFBI). Collagens contribute to tumor progression, metastasis formation, and modulation of the therapeutic response. Elastin, through elastosis, can facilitate changes that favor tumor progression. Laminins increase cancer cell resistance to apoptosis and stimulate tumor development through interactions with other proteins and cytokines. Fibronectin promotes cancer cell proliferation. LOX overexpression in tumor tissue and excessive collagen production enhance cell adhesion, migration, and invasiveness. A deeper understanding of ECM proteins in modulating the TME could serve as a foundation for developing novel therapeutic strategies to overcome drug resistance in cancer [21,22].

The information above highlights the significance of drug resistance and the ongoing extensive research on its development mechanisms. The history of cancer treatment goes back many years. Many different treatment methods have been modified and improved. New ones have emerged, such as the advent of targeted therapies, but surgery remains the same. Despite this, oncological treatment still has its limitations [2]. That’s why exosomes have attracted scientists’ attention. A relatively recent yet rapidly growing area of research focuses on exosomes, which play a key role in maintaining cellular homeostasis, clearing cellular debris, and facilitating intercellular communication [23]. Moreover, they regulate various processes, including immune responses, cancer development, tissue regeneration and progression of neurodegenerative diseases [5,24,25].

**Figure 1:**
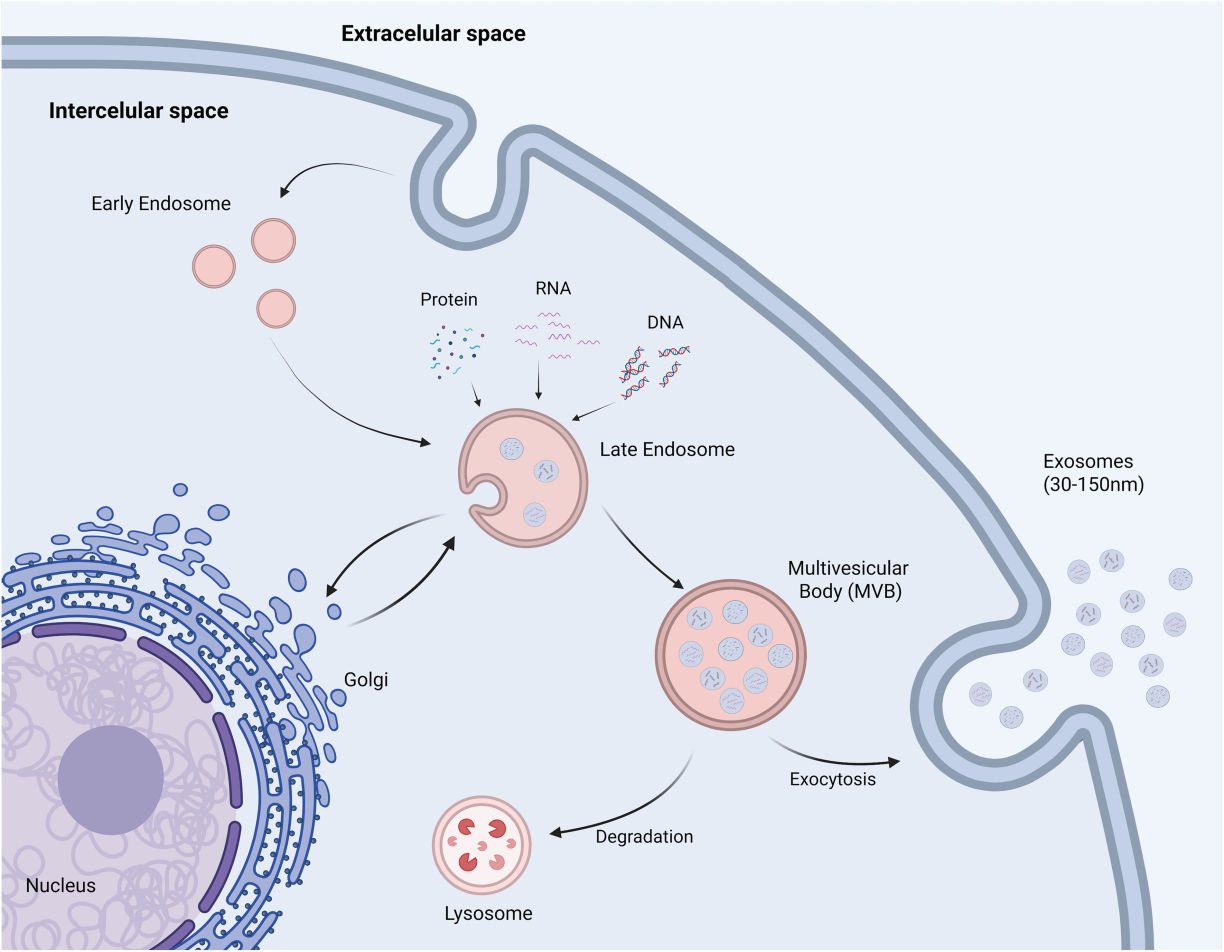
Biogenesis of exosomes. As a result of endocytosis, during which the plasma membrane becomes invaginated into the cell interior, vesicles called early endosomes are formed. As they mature, early endosomes transform into late endosomes, in which the membrane begins to fold and invaginate into their interior locally. This way, intraluminal vesicles (ILVs) are formed, into which selectively selected molecules such as proteins, lipids, RNA, or DNA are placed. Structures containing numerous ILVs are called multivesicular bodies (MVBs) and play a key role in further sorting of cellular cargo. MVBs can then fuse with the lysosome, leading to degradation of their contents, or fuse with the plasma membrane, releasing ILVs into the extracellular space as exosomes (Created in BioRender, accessed on 10 October 2024)

Exosomes have emerged as a significant area of research in cancer drug resistance because, unlike classical mechanisms, they show that cancer cells can “talk” to each other and their environment. They facilitate the transfer of miRNAs and proteins that confer a resistant phenotype to otherwise sensitive cells [26]. Exosomes are a subclass of bilayer membrane vesicles within the broader category of extracellular vesicles (EVs) [27]. They typically range in size from 30 to 150 nm [28,29]. They originate through invaginations within the endosomal compartment, subsequently forming multivesicular bodies (MVBs), which are released into the extracellular space [30,31]. The process of exosome formation is presented in Fig. 1.

This review aims to explore the role of exosomes in cytotoxic resistance mechanisms and their potential as both diagnostic and therapeutic tools in cancer treatment.

## 2 Exosomes and Their Composition in the Context of Cytostatic Resistance

Exosomes are extracellular vesicles that mediate intercellular communication. Their composition is highly diverse, as they can contain proteins, lipids, DNA, and various forms of RNA, including mRNA and ncRNA. Exosomes secreted by cancer cells are referred to as tumor-derived exosomes (TEX) [25]. Studies have demonstrated that TEX modulate chemotherapy sensitivity by transferring a resistance phenotype to target cells [23].

One example concerns the P-gp protein. P-gp is a key factor in developing MDR in cancer cells [18]. Lopes-Rodrigues et al. were the first to demonstrate that vesicles secreted by cancer cells contain P-gp [32]. As a transport protein, it facilitates the efflux of chemotherapeutic agents such as paclitaxel (PTX), doxorubicin (DOX), and mitoxantrone from cancer cells. Consequently, their intracellular concentration decreases, leading to drug resistance [33,34]. Ovarian cancer (OC) data showed that exosomes from platinum-resistant A2780 cells contain higher P-gp than those from sensitive A2780, consistent with an exosomal role in MDR [35]. In turn, another study showed that human breast cancer cells are resistant to adriamycin (MCF7/ADM) secreted exosomes carrying P-gp proteins and ubiquitin carboxyl terminal hydrolase-L1 (UCH-L1) to the extracellular microenvironment. Exosomes interacted with adriamycin-sensitive human breast cancer cells (MCF7/WT), facilitating the transfer of a chemotherapy-resistant phenotype [36]. The researchers reached a similar conclusion regarding osteosarcoma and the DOX drug [37].

BCRP is another key factor contributing to the development of drug resistance. BCRP belongs to the ATP-binding cassette (ABC) family and is a defense mechanism against toxins and xenobiotics. It facilitates the excretion and restricts the absorption of potentially toxic substrates, including various chemotherapeutic agents used in cancer treatment [38]. Many sources indicate its essential role in shaping drug resistance [39–41]. Notably, Kong et al. were the first to suggest that BCRP is associated with and secreted via exosomes [42]. However, the preliminary observations by Kong et al., suggesting that BCRP is associated with and secreted via exosomes, require cautious interpretation, as the findings still need further validation and independent confirmation in different experimental models. It should be emphasized that this study was exploratory in nature, and the applied methodologies had limited capacity to demonstrate the interaction between BCRP and exosomes unequivocally. A significant interaction between BCRP exosomes and resistance development can be found in the studies of Kim et al. This study based on cell lines derived from triple-negative breast cancer (TNBC), examined BCRP and MDR1 and MRP1. MDR1, MRP1, and BCRP expression in extracellular vesicles (EVs) derived from breast cancer cells increased after chemotherapy *in vitro*, particularly in drug-resistant cell lines [43].

Another exosomal protein in drug resistance is programmed death-ligand 1 (PD-L1). PD-L1 is a membrane-bound ligand expressed on the surface of various cancer cells and exosomes, which is referred to as exosomal PD-L1 [44]. In esophageal squamous cell carcinoma (ESCC), elevated PD-L1 expression has been correlated with advanced clinical stage, poor prognosis, and resistance to chemotherapy [45,46]. These findings are further supported by *in vitro* studies showing that the paclitaxel-resistant EC-9706R cell line exhibits higher levels of exosomal PD-L1 than its paclitaxel-sensitive parental counterpart. Moreover, exosomes derived from EC-9706R cells enhanced invasion, migration, and resistance to paclitaxel in EC-9706 cells, potentially through regulation of the signal transducer and activator of transcription 3 (STAT3)/miR-21/phosphatase and tensin homolog (PTEN)/protein kinase B (AKT) axis [47]. Exosomal PD-L1 has also been detected in various cancers, including melanoma [48], breast cancer [49], glioma [50], stomach cancer [51], and lung cancer [52]. Moreover, exosomal PD-L1 plays a crucial role in enabling cancer cells to evade immune surveillance. PD-L1 binds to the programmed cell death protein 1 (PD-1) receptor on T cells, inhibiting their activity, ultimately suppressing the immune response [53].

Among the immunosuppressive molecules transported by exosomes, prostaglandin E2 (PGE2) and transforming growth factor beta (TGF-β) play a crucial role in conferring resistance to cytostatics by reducing cytotoxicity and promoting an immunosuppressive TME [25]. PGE2 activates signalling pathways such as PI3K/Akt and mitogen-activated protein kinase (MAPK) essential for cancer cell survival, proliferation, and chemotherapy resistance [54,55]. In the late stages of cancer, TGF-β promotes tumor progression by upregulating vascular endothelial growth factor (VEGF) expression [56]. Xiang et al. provided evidence supporting the immunosuppressive properties of exosomal PGE2 and TGF-β in cancer. They demonstrated that T cell-derived exosomes can induce myeloid-derived suppressor cells (MDSCs) accumulation in tumors. Moreover, tumor-derived exosomal PGE2 and TGF-β contribute to MDSC accumulation [57]. Additionally, studies have shown that exosomes derived from specific cancer cells can induce fibroblast differentiation into myofibroblasts. It was established that exosomally transported TGF-β is responsible for this differentiation. TGF-β, through activation of the SMAD family member 3 (SMAD3) signaling pathway, leads to the differentiation of fibroblasts into myofibroblasts [58].

A crucial mechanism underlying drug resistance in cancer cells is the transfer of non-coding RNAs (ncRNAs), including miRNAs and circular RNAs (circRNAs), via TEX [28]. The development of cancer therapies based on miRNAs, short hairpin RNAs (shRNAs), and small interfering RNAs (siRNAs) began in the early 21st century [2]. miRNAs are small, non-coding RNA molecules of 17–24 nucleotides in length, which maintain a high degree of conservatism. They act as gene regulators by binding to the 3^′^ untranslated region (3^′^ UTR) sequences of mRNA, inhibiting their expression [59]. They act as regulators of many key cellular processes, controlling several essential functions, including cell signaling, inflammatory processes related to cancer development, T cell and stem cell differentiation, and maintaining metabolic homeostasis [60]. TEX contribute to oncogenesis by influencing neighboring immune cells by transporting miRNAs, among other biomolecules. miRNAs that promote tumor growth are called oncomiRs [61]. Table 1 presents examples of miRNAs and their influence on drug resistance.

**Table 1:** Role of noncoding RNAs in drug resistance

Exosomal RNA	Tumor types	Function in the development of drug resistance	References
**miR-1246**	OC	Regulation of the Cav1/p-gp/M2 pathway, inhibiting Cav1 expression and increasing p-gp (MDR1) levels, which promotes paclitaxel resistance high	[60]
	PC	Expression of miR-1246 downregulated CCNG2 levels	[73]
**miR-135b**	CRC	Increases resistance to oxaliplatin Reduces cell apoptosis in CRC cells Promotes cetuximab resistance	[69] [74] [75]
**miR-135b**	GC	Increase cisplatin (CIS) resistance in gastric cancer	[70]
**miR-135b**	PC	Promotes gemcitabine resistance in cells	[71]
**miR-1229-3p**	GC	Overexpression of miR-1229-3p induced significant chemoresistance to 5-fluorouracil (5-FU), up-regulation of thymidylate synthase (TS) and dihydroprimidine dehydrogenase (DPD) and down-regulation of SLC22A7	[76]
**miR-21**	NBL ESCC	Promotes chemoresistance through a novel exosomal miR-21/TLR8-NF-кB signaling STAT3/miR-21/PTEN/Akt pathway, resulting in greater resistance to paclitaxel	[77] [47]
**miR-223**	EOC	Promotes drug resistance of EOC cells via the PTEN-PI3K/AKT pathway	[78]
**miR-365**	PDAC	Induction of gemcitabine resistance	[79]
**miR-196**	HNC	Confer CIS resistance by targeting CDKN1B and ING5	[80]
**miR-493**	GC	Involved in chemoresistance to paclitaxel by downregulating MAD2L1	[81]
**miR-146a-5p**	UBC	Overexpression promoted CAF-UBC cell interactions, cancer stemness, and chemoresistance to gemcitabine and CIS treatment	[82]
**miR-433**	OC	Promotes resistance to paclitaxel through the induction of cellular senescence	[83]
**miRNA-20a**	LC	Promoted NSCLC cell proliferation and resistance to CIS (DDP) and suppressed the PTEN/PI3K-AKT pathway to encourage the progression and chemoresistance	[84]
**circ_0006174**	CRC	Promotes DOX resistance via mediating the miR-1205/CCND2 axis	[85]
**circ_0001610**	CRC	Promotes oxaliplatin resistance by promoting oxidative phosphorylation (OXPHOS)	[86]
**circ_0000338**	CRC	Enhances 5-fluorouracil resistance by regulating miR-217 and miR-485-3p	[87]
**circBIRC6**	PDAC	CircBIRC6 enhances oxaliplatin resistance via regulating the non-homologous end joining (NHEJ) dependent DNA repair	[88]
**circBACH1**	BC	Paclitaxel-treated BC-derived exosomes (PTX-EXO) promote PTX-resistance and angiogenesis through upregulation circBACH1	[89]

Note: OC, Ovarian cancer; PC, Pancreatic cancer; OSCC, Oral squamous cell carcinoma; CRC, Colorectal cancer; GC, Gastric cancer; BC, Breast cancer; RCC, Renal cell carcinoma; GC, Gastric cancer; ESCC, Esophageal squamous cell carcinoma; EOC, Epithelial ovarian cancer; PDAC, Pancreatic ductal adenocarcinoma; HNC, Head and neck cancer; UBC, Urothelial bladder cancer; LC, Lung Cancer; CRC, Colorectal cancer.

Jin et al. demonstrated the presence of exosomal miRNAs in the serum of patients with advanced colorectal cancer. The key exosomal miRNAs associated with chemoresistance included miR-21-5p, miR-1246, miR-1229-5p, and miR-96-5p [62]. For instance, miR-1246 can be transported via exosomes derived from OC cells. Oncogenic miR-1246 contributes to chemoresistance by suppressing caveolin-1 (Cav1) expression, a protein essential for forming caveolae-specialized membrane structures involved in cellular transport and signaling. In addition, exosomal miR-1246 promotes tumor development in the TME by acting on M2 macrophages, which promote oncogenic progression [60]. miR-1246 was also detected in exosomes from breast cancer [63], squamous cell carcinoma of the oral cavity [64] and glioma [65]. In the study by Mikamori et al., the authors demonstrated that exosomes play a key role in regulating miRNA levels and intercellular communication in PDAC. The study showed that the miR-155 loop confers chemoresistance in these cells. The mechanism was as follows: (1) prolonged exposure to gemcitabine (GEM) increases miR-155 expression in PDAC cells; (2) elevated miR-155 promotes both exosome secretion and anti-apoptotic activity, contributing to chemoresistance; (3) exosomes deliver miR-155 to other PDAC cells, propagating the resistance effect [66].

Many findings suggest exosomes can also release miRNA from tumor-associated macrophages (TAMs) [67]. Exosomes from TAMs carry, among others: mi365, miR-21, miR-223, miR-1246, miR-196 [68]. Conversely, exosomal miR-135b has been implicated in chemoresistance in colorectal cancer (CRC), gastric cancer (GC), and pancreatic cancer (PC) [69–71].

It has been proven that exosomal circRNAs also impact the development of chemoresistance. CircRNAs are a class of circular RNAs characterized by covalently closed-loop structures. They arise due to alternative splicing of exons or introns of genes [72]. The influence on chemoresistance is very often connected to the miRNA-RNA axis. Table 1 also presents examples of circRNA and their impact on drug resistance.

The use of small RNA-based therapies is associated with several challenges, the most critical being the inability to elicit clinically significant anticancer activity in clinical trials [2].

## 3 Exosomes and Intercellular Interactions

### 3.1 Effect of Exosomes on Neighboring Cells

Exosomes are present in various body fluids and are crucial in transferring biological materials from donor to recipient cells. Consequently, they are essential for intercellular communication, influencing the regulation of both physiological and pathological microenvironments [62]. Recently, extensive research has focused on the tumor TME, a critical factor in the development of chemoresistance. TEX influence neighboring cells, including fibroblasts, endothelial cells, and immune cells. Intercellular communication in the TME is also ensured by exosomes delivered by various microenvironment cells, including fibroblasts, macrophages, endothelial cells, adipocytes and pericytes [90].

Cancer-associated fibroblasts (CAFs) are key components of TME surrounding cancer cells. CAFs exhibit sustained activity, characterized by continuous stimulation of cellular metabolism, including collagen and elastin production and enzyme secretion. Sustained activation of fibroblasts in cancer tissue supports intensive proliferation and development of cancer cells. However, CAFs are abundant yet remain an insufficiently understood cell type in the TME, capable of promoting and inhibiting tumor growth, depending on the conditions and organ type [91]. The promotion of tumor progression by CAF has been confirmed, among others, in the case of liver cancer [91] or breast cancer [92]. CAFs promote the development of drug resistance in many processes, e.g., CAFs contribute to drug resistance through various mechanisms, such as inducing fibrous hyperplasia, which can directly lead to decreased drug perfusion, thereby reducing the effect of chemotherapy [93]. In another study, pancreatic stellate cells (PSCs) were shown to protect pancreatic ductal adenoma (PDAC) from the effects of gemcitabine. This suggests that CAFs within tumor tissues reshape the TME, fostering chemotherapy resistance [94]. The role of CAFs in tumor invasion and metastasis is becoming increasingly better known and described [95,96]. However, the involvement of exosomes in the development of cytoresistance based on the TME is a relatively new research trend. CAF-derived exosomes in PDAC, although not intrinsically resistant to gemcitabine, have been shown to promote chemoresistance by maintaining signaling with cancer cells during treatment. CAFs deliver exosomes containing miR-3173–5p affecting acyl-CoA synthetase long-chain family member 4 (ACSL4) secretion and inhibiting ferroptosis [93]. In research on chemoresistance in GC, CAFs have been shown to secrete exosomal miR-522, suppressing ferroptosis in GC tumor cells by targeting ALOX15 and preventing lipid-ROS accumulation, contributing to chemotherapy resistance. Another study demonstrates the interaction between CAFs and urothelial bladder cancer (UBC) cells. CAF-derived miR-146a-5p facilitates the formation and maintenance of the cancer stem cell (CSC) niche, promoting CSC-associated survival and invasiveness. CAFs interact with cancer cells through direct contact, such as cell adhesion mediated by SVEP1. Intercellular communication also occurs through regulating ARID1A and PRKAA2 gene expression in UBC cells, activating the signal transducer and activator of transcription 3 (STAT3) and mechanistic target of rapamycin (mTOR) signaling pathways. STAT3 activation enhances the expression of CSC markers, including SRY-box transcription factor 2 (SOX2) cluster of differentiation 44 (CD44), and integrin β1 [82].

Numerous studies suggest that the vascular endothelial cells, as a key component of the TME, play a crucial role in metastasis formation [97]. Angiogenesis, the process of forming new blood vessels, is closely associated with tumor development. Various tumor cell types secrete exosomes enriched with proangiogenic proteins, thereby promoting angiogenesis. Proangiogenic factors detected in exosomes include vascular endothelial growth factor (VEGF) transforming growth factor-beta (TGF-β) interleukin-6 (IL-6), interleukin-8 (IL-8), tissue inhibitor of metalloproteinases-1 (TIMP-1), and metalloproteinases-2 (TIMP-2) in glioblastoma multiforme; VEGF, basic fibroblast growth factor (bFGF), MMP9, and hepatocyte growth factor (HGF) in multiple myeloma; IL-6, VEGF, and MMP2 in melanoma; endothelin-1, IL-8, Thrombospondin 2 (TSP-2), urokinase-type plasminogen activator (uPA), and VEGF in lung adenocarcinoma; EGF-like repeats and discoidin I-like domain-3 (EDIL-3) in bladder cancer; and annexin II in breast cancer [98,99]. Additionally, tumor endothelial cells (TECs) not only stimulate the secretion and expression of proangiogenic molecules but also facilitate tumor metastasis. TECs show some variability depending on their origin and differ from normal endothelial cells (NECs) in their response to proangiogenic factors such as EGF, adrenomodulin, and VEGF. Autocrine VEGF secreted by TECs enhances their survival and promotes migration. Another key characteristic of TECs is their drug resistance [100].

As previously mentioned, exosomes derived from cancer cells and different cells associated with cancer can carry a variety of bioactive molecules, including proteins, lipids, and different types of RNA, such as miRNAs. These miRNAs can modulate endothelial cell function, influencing their migration, proliferation, and angiogenic potential, thereby promoting tumor progression. Exosomes derived from lung cancer tissue have been shown to selectively deliver miRNA-210 to endothelial cells, thereby promoting tumor angiogenesis [101]. Another study demonstrated higher expression of miR-92a in exosomes derived from chronic myeloid leukemia (CML) cells compared to endothelial cells. This resulted in enhanced cell migration and capillary formation [102]. In turn, Keklikoglou et al. demonstrated that taxanes and anthracyclines, two classes of cytotoxic drugs, induce increased exosome secretion from breast cancer cells, contributing to metastasis. Exosomes produced during chemotherapy are enriched in annexin-A6 (ANXA6), a Ca^2+^-dependent protein that promotes nuclear factor κB (NF-κB)-dependent activation of endothelial cells, induction of Chemokine (C-C motif) Ligand (CCL2), and expansion of Ly6C^+^CCR2^+^ monocytes, facilitating the development of lung metastases [103]. Disruption of tight junctions between endothelial cells increases vascular permeability, facilitating tumor cell extravasation and metastasis. Vascular permeability is also enhanced by the delivery of miRNAs through exosomes derived from cancer cells. In the study by Liu et al., exosomal miR-29a was shown to originate from epithelial-mesenchymal transition (EMT) in CRC cells. Exosomal miR-29a was transported to endothelial cells within the TME, increasing vascular permeability by downregulating the expression of intercellular junction proteins, including Zonula occludens-1 (ZO-1), Claudin-5, and Occludin. As a result, increased vascular permeability promotes the development and metastasis of CRC [104]. In turn, metalloproteinase 17 (ADAM17), present in circulating exosomes from CRC patients, may promote metastasis. ADAM17 affects vascular endothelial cells, increasing vascular permeability by modifying cadherins in the cell membrane [105].

Additionally, exosomes can influence the TME by modulating the immune response through their impact on immune cells, including macrophages and lymphocytes. This can suppress the immune response against tumor cells, thereby promoting tumor progression and therapy resistance. Immune cells produce various cytokines that negatively regulate immune function and promote tumor growth. Among these, TAMs are the most abundant [106]. TAMs originate from monocytes and represent the dominant immune cell type in the TME, comprising 15%–20% of the tumor mass. Macrophages differentiate into two main phenotypes. M1 macrophages exhibit a pro-inflammatory function, supporting the immune response and restricting tumor growth. Stimulated by T helper 1 (Th 1) cytokines, such as interferon gamma (IFN-γ) and lipopolysaccharide (LPS), which activate Toll-like receptors (TLRs) to promote inflammation. In contrast, M2 macrophages exhibit anti-inflammatory properties, promoting tumor progression, angiogenesis, and metastasis through stimulation by Th2 cytokines, including interleukin-4 (IL-4), IL-10, and TGF-β [107,108]. M2 macrophages contribute to cancer metastasis by secreting various enzymes in a paracrine manner. These enzymes degrade collagen components in the ECM, leading to structural breakdown and facilitating cancer cell migration [108]. Extensive research has documented the role of macrophages, particularly M2, in metastasis, angiogenesis, and tumor survival [109–111].

While interactions between cancer cells and M2 macrophages have been extensively studied, the mechanisms underlying M2 macrophage activation by cancer cells remain a topic of interest for researchers and clinicians. Exosomes, as key mediators of intercellular communication, may play a crucial role in this process. Exosomes targeting macrophages contribute to the formation of a metastatic niche, thereby complicating patient treatment. Zhao et al. demonstrated elevated expression of miR-934 in CRC, including colorectal cancer liver metastases (CRLM). They demonstrated that exosomal miR-934 induces M2 macrophage polarization by downregulating phosphatase and tensin homolog (PTEN) expression and activating the PI3K/AKT signaling pathway. These findings highlight the role of TEX in mediating interactions between tumors and TAMs within the metastatic microenvironment, playing a crucial role in CRLM [112]. Similarly, exosomal FGD5-AS1 from PC cells can drive macrophage polarization toward the M2 phenotype by activating the STAT3/NF-κB pathway. This mechanism involves the interaction of FGD5-AS1 with the p300 protein, which leads to STAT3 acetylation and, consequently, its translocation to the nucleus and an increase in the transcriptional activity of the STAT3/NF-κB pathway [113]. However, increased levels of exosomal miR-519a-3p were detected in serum in GC patients with liver metastases (LM). Exosomal miR-519a-3p initiates the MAPK/extracellular signal-regulated kinase (ERK) pathway activation by regulating DUSP2, which leads to macrophage polarization toward the M2 phenotype. Such polarized macrophages support the development of gastric cancer liver metastases (GC-LM) by stimulating angiogenesis and creating an intrahepatic premetastatic niche [114]. Exosomes derived from cholangiocarcinoma (CCA) containing LINC01812 play a key role in macrophage polarization toward the M2 phenotype. This process promotes perineural invasion (PNI) and tumor progression. LINC01812 interacts with the TUBB4B protein to activate the Notch signaling pathway in macrophages, leading to increased secretion of the chemokine CCL2. This LINC01812-Notch-CCL2 signaling axis promotes cancer cell migration and invasion. Additionally, LINC01812-containing exosomes can influence neuronal cells by enhancing their secretion of neurotrophic factors, further promoting PNI [115].

TAMs promote tumor immune evasion by recruiting immunosuppressive regulatory T cells (Tregs), thereby attenuating the cytotoxic activity of effector lymphocytes and weakening the host antitumor immune response [116]. Exosomes released from TAMs play a pivotal role in this process. Zhou et al. reported that microarray profiling of TAM-derived exosomes in epithelial ovarian cancer (EOC) identified enrichment of specific miRNAs, including miR-29a-3p and miR-21-5p. Transfection of these miRNAs into CD4+ T cells directly inhibited STAT3 signaling and disrupted the Treg/Th17 balance, synergistically affecting STAT3 suppression. These findings highlight that TAM-derived exosomes mediate crosstalk with T cells to establish an immunosuppressive tumor microenvironment, ultimately fostering tumor progression and metastasis [117]. Additionally, CD4+ T helper (Th) cells, which secrete IL-4, IL-13 and IL-10-producing Tregs, play a key role in macrophage polarization [118]. Moreover, Li et al. demonstrated that exosomal LINC01232 facilitates tumor immune evasion. TAMs secrete exosomal LINC01232, which promotes immune evasion by tumor cells. This mechanism involves direct binding of LINC01232 to E2F2, facilitating nuclear transport and jointly stimulating NBR1 gene transcription. Enhanced interaction between NBR1 and ubiquitinated major histocompatibility complex class I (MHC-I) via the ubiquitin domain promotes MHC-I degradation in autophagolysosomes. Consequently, this reduces MHC-I levels on the tumor cell surface, enabling immune evasion from CD8+ cytotoxic T lymphocytes (CTLs) [119]. Additionally, within the TME, cancer cells express PD-L1 on their surface, binding to PD-1 on T lymphocytes, inhibiting their ability to destroy tumor cells [110].

### 3.2 Exosomes and Inflammatory Processes

Exosomes may play a crucial role in inducing and modulating TME inflammation, thereby influencing cytostatic resistance development. Inflammation, both extratumoral (triggered by external factors such as infections or obesity) and intratumoral (driven by cancer mutations), plays a pivotal role in tumor progression. Inflammation and tumorigenesis share common signaling pathways and key regulatory molecules involved in apoptosis, proliferation, and angiogenesis. Chronic inflammation may promote tumor initiation, progression, and invasiveness by releasing bioactive proinflammatory factors that shape the TME [120,121]. Key proinflammatory factors associated with cancer include interleukin (IL)-1β, IL-6, and tumor necrosis factor alpha (TNF-α), along with the transcription factors NF-κB and STAT3 [121]. Numerous studies have confirmed the link between inflammation and cancer [122–124]. Thus, the role of exosomes in this process remains an active area of research.

Exosomes regulate inflammation, in part, by activating TLRs on the surface of monocytes. Bretz et al. isolated exosomes from malignant ascitic fluid in patients with OC. TEX were internalised by THP-1 monocytes, stimulating the secretion of proinflammatory cytokines, including IL-1β, IL-6, and TNF-α. This mechanism involved exosomal interaction with TLR2 and TLR4 receptors, activating signaling pathways that induced NF-κB and STAT3 [125]. Haderk et al. demonstrated that TEX carrying noncoding RNAs, such as hY4, promote an inflammatory microenvironment and facilitate immune evasion by inducing PD-L1 expression. Exosomal transfer from chronic lymphocytic leukemia (CLL) cells to monocytes enhanced the secretion of cytokines, including CCL2, CCL4, and IL-6, while upregulating PD-L1 expression. This mechanism was TLR7-dependent, and its inhibition abrogated these effects [126].

The interaction between TEX and macrophages plays a crucial role in immune responses and inflammatory processes. Macrophages, essential for antigen presentation, phagocytosis, and immunomodulation, exhibit remarkable functional plasticity. The microenvironment shapes their phenotype, enabling them to perform diverse functions in inflammation [121]. GC-derived exosomes have been shown to activate NF-κB, stimulating the production of inflammatory cytokines, including G-CSF, IL-6, IL-8, IL-1β, CCL2, and TNF-α, thereby inducing a pro-inflammatory M1 macrophage response [127,128]. Furthermore, the interaction between breast cancer-derived exosomes and macrophages largely depends on TLR2 expression [127]. Exosomal proteins can also drive inflammation, with Annexin A2 (AnxA2) being a key example. TEX containing AnxA2 activate M1 macrophages, triggering NF-κB, STAT3, and p38 MAPK signaling pathways, thereby enhancing the secretion of proinflammatory cytokines, including IL-6 and TNF-α [129].

In the context of tumor exosome-mediated inflammation, it is essential to consider their impact on dendritic cells. Although the mechanisms by which TME and exosomes influence dendritic cells to promote tumorigenesis remain insufficiently understood. Dendritic cells are specialized antigen-presenting cells (APCs) responsible for recognizing, processing, and presenting antigens to T lymphocytes. This process is mediated by major histocompatibility complex (MHC) molecules, co-stimulatory proteins, and cytokines, ultimately leading to immune activation. Exosomes can be potent suppressors of immunity by inhibiting dendritic cell differentiation [121]. Additionally, TEX can activate dendritic cells, leading to increased production of inflammatory mediators, including IL-6 and prostaglandin E1 (PGE1), which subsequently enhances cancer cell invasion and metastasis [130].

MDSCs constitute a heterogeneous population of immature myeloid cells that play a crucial role in immune suppression, particularly in cancer. Under physiological conditions, MDSCs differentiate into monocytes, neutrophils, and dendritic cells. However, in the TME, exposure to proinflammatory cytokines and growth factors disrupts their maturation, resulting in the acquisition of an immunosuppressive phenotype [131]. However, in the TME, exposure to proinflammatory cytokines and growth factors disrupts their maturation, resulting in the acquisition of an immunosuppressive phenotype [132]. Conversely, glioma-derived exosomes carrying miR-10a or miR-21 are internalised by MDSCs, enhancing immunosuppression by increasing arginase activity, generating reactive oxygen species (ROS) and nitric oxide (NO), and secreting immunosuppressive cytokines [133].

The data mentioned above highlight the fundamental role of exosomes as mediators of intercellular communication, which is pivotal in developing resistance to cancer treatment. In summary, cancer cell-derived and tumour microenvironmental cell exosomes contribute to chemoresistance by transferring genetic material, modulating the immune response, and amplifying survival-related signalling pathways.

## 4 Exosomal Biomarkers

As previously mentioned, exosomes carry many types of molecules, such as proteins, lipids or RNAs [134]. Understanding what exosomes transport and under which conditions is crucial, as intercellular communication via exosomes is highly selective and not random. Therefore, determining exosomal content opens the possibility of identifying biomarkers that are valuable for diagnostics and guiding treatment strategies for various diseases, including cancer [134–136].

In recent years, numerous clinical trials have been conducted using exosomes, with approximately 50% of cancer therapy trials focusing on exosome biomarkers. Because exosomes are easily available in many biofluids, they offer a convenient source for biomarker testing [134,137,138]. Currently, the liquid biopsy is the most commonly used method, as it is minimally invasive [139]. Nonetheless, further validation is required, and exosome-derived biomarkers must be compared with established cancer biomarkers [140].

Exosomal miRNAs are considered one of the most abundant and stable molecules within exosomes, making them prime candidates as exosomal biomarkers [141]. Compared to normosomes, exosomal miRNAs originating from cancerous sources show a 30-fold increase in concentration, underscoring their diagnostic potential. This biomarker group is highly diverse, varying by cancer type and exosome source [139].

Among numerous miRNAs, potentially universal exosomal biomarkers include: iR-29a, miR-141-3p, miR-375, miR-106b-3p, miR-221/222, miR-200b-3p, miR-18a, miR-20b, miR-1307-5p, miR-519a-3p, miR-379-5p, and miR-410-3p [139]. In invasive submucosal colorectal cancer, biomarkers such as miR-181, miR-193 b, miR-195, and miR-411 have been identified and validated alongside previously established circulating cell-free serum (cf-miRNA) markers, demonstrating significant diagnostic performance in detecting lymph node metastasis [141]. Specifically, in colorectal cancer, miR-29a, miR-92a-3p, miR-128-3p promote metastasis, with miR-128-3p notably overexpressed at advanced tumor stage (III–IV) [139]. Additionally, exosomal miR-126, miR-1290, miR-23a, and miR-940 have emerged as promising diagnostic biomarkers in colorectal cancer, aiding in metastasis stage classification. Another study showed the downregulation of exosomal miRNA, such as miR-99b-5p and miR-150-5p, in CRC patients compared to healthy controls. Next, in esophageal cancer, exosomal miR-93-5p is believed to promote the proliferation of esophageal squamous carcinoma cells by suppressing the expression of p21 and cyclin D1 [138].

In breast cancer, exosomal miRNAs are used for cancer subtyping and setting a stemness and metastatic properties [140]. Scientists showed higher exosomal miR-101 and miR-372 levels in the serum of breast cancer patients compared to controls, highlighting miR-373’s potential as a breast cancer biomarker [135]. Several ovarian exosomal biomarkers have also been proposed for cancer diagnosis and monitoring disease progression. For example, the Lutgendorf group showed 22 exosomal RNA biomarkers strongly correlated with the progression class of OC [142]. In tissue sarcoma, miR-25-3p, miR-92a-3p, miR-34a, MT1-MMP, and MMP-14 have been implicated in disease progression [140]. Very valuable research was conducted by Gotanda et al., demonstrating that exosomal miR-328 could serve as a biomarker for assessing BCRP activity in the human intestines, because miR-328 negatively regulates BCRP expression [143]. Furthermore, exosomal miR-1246 in the body fluid of glioma patients has shown potential for monitoring glioma recurrence [65] and may also serve as a diagnostic biomarker for gastrointestinal cancers [144].

Exosomal circRNAs influence cancer proliferation, invasion, and drug resistance [145]. They represent excellent diagnostic and prognostic biomarkers for liquid biopsy because of their stability, long half-life, resistance to degradation, and broad distribution in many types of cancer [146]. In colorectal cancer, circRNAs such as circ-133 and circPABPC1 promote metastasis. Furthermore, analysis showed that these circRNAs can react with various miRNAs, enhancing metastatic foci formation [139]. For instance, hypoxic tumor-derived exosomal circ-133 promotes development of EMT in CRC through the miR-133a/GEF-H1/RhoA axis [146]. Esophageal squamous cell carcinoma is characterized by elevated levels of circRNAs hsa-circ-0001946 and hsa-circ-0043603, making them potential biomarkers of this cancer also [138]. Additionally, serum exosomes circMYC overexpression in nasopharyngeal carcinoma corelates with tumor size, lymph node metastasis, and survival rate [146].

Long non-coding RNAs (lncRNAs) have also emerged as promising biomarkers for cancer detection [139], as they may mediate cancer cell migration and invasion. Comparative analysis of exosomal RNA from CRC patients and healthy serum revealed 569 upregulated and 475 downregulated lncRNAs. For example, lncRNA UCA1 was overexpressed in the advanced stage of cancer, similar to lncRNA SPRY4-IT1. While the levels of lncRNA PVT1/VEGFA and LINC00161 were distinctly inverted [139]. In CRC patients, serum exosomal lncRNA FOXD2-AS1 has also demonstrated promising diagnostic potential [147]. In oral squamous-cell carcinoma (OSCC), it was shown that FOXD2-AS1 negatively regulated miR–185–5p, rather than the whole PLOD1/Akt/mTOR pathway activity, promoting cancer growth, invasion and migration [148]. Arima et al. reported that exosomal lncRNA brain cytoplasmic RNA 1 (BCYRN1) expression was significantly higher in bladder cancer patients than healthy individuals, contributing to enhanced proliferation, migration and invasion of cancer cells [149]. The lncRNAs have impressive potential to be biomarkers in gastric cancer (GC) because they are easy to detect in the fluids of the body. The studies showed some lncRNAs, such as LINC00152 or CASC15, to be crucial for tumour invasion, and low survival of GC patients [150].

It is important to note that exosomes also transport proteins that may serve as potential biomarkers. Despite their heterogeneity, exosomal proteins from diverse origins remain reliable prognostic, diagnostic, and predictive biomarkers in cancer treatment. For example, in colorectal cancer, the ADAM17 protein, which cleaves the E-cadherin junction, has enhanced vascular permeability and promotes metastasis [105]. Other potential protein biomarkers, such as HSPC111, Cyr61, and FMNL2, activate metabolic pathways, including XCL5-CXCR2 axis, αV β5/FAK/HIF-1α/STAT3/MMP2 pathway, and EGFL6/CKAP4/ERK axis, facilitating metastasis. In the case of hepatocellular cancer, exosomal protein biomarkers include CTLA-4, which activates the PTEN/CD44 signal pathway, and LOXL4, activating the FAK/Src pathway [139]. Barhoum group showed that exosomal protein MMP14 concentration is significantly higher in patients with adenoma vs. control patients [151]. Another protein, SUMO-specific protease 1 (SENP1), may serve as a prognostic biomarker for melanoma, influencing overall survival [152]. The other research group analysed lymphocyte migration regulation-related proteins (WASL, STK10 and WNK1) in the urine exosomes of lung cancer because of their non-exosomal analogous significant function in cancer pathogenesis. Finally, they demonstrated the tested proteins as potential lung cancer biomarkers [153]. In nasopharyngeal carcinoma, exosomal phosphatase and tensin homolog (PTEN) has been detected, which may assist in diagnosis and treatment planning for that type of cancer, as it plays a crucial role in radiotherapy and immunotherapy efficacy, tumour growth, and the immune microenvironment [154].

Importantly, some studies showed that exosomes can also inhibit cancer progression. Exosomes can convey biomarkers of cancer metastasis inhibition, such as miR-140-3p (which upregulate the expression of BCL9 and BCL2) and circFNDC3B (which inhibit miR-937-5p and upregulate TIMP3) in colorectal cancer [155,156]. In PC, miR-485-3p reduces PAK1 expression, while miRNA-339-5p decreases TGFBR3 level [139]. However, such reports are in the minority, and most indicate a positive correlation with the potential for cancer development. Nevertheless, there is no doubt that the identification of new exosomal biomarkers is crucial in modern medicine, but it remains challenging because of the complicated and yet non-standardised protocol of exosome isolation. Exosomes are secreted by all cell types, both healthy and cancerous. They mix in body fluids, making it difficult to assign a specific biomarker to a specific disease. The content of exosomes depends, among other things, on the physiological state of the organism and environmental conditions, which are variable. All this makes the analysis of exosomes very difficult. Therefore, further studies are necessary to assess exosomal cargos as early diagnostic biomarkers [151,157].

## 5 Therapeutic Strategies Involving Exosome Engineering

Exosomes have emerged as promising candidates for therapeutic applications in oncology due to their intrinsic ability to mediate intercellular communication by transferring proteins, lipids, and nucleic acids. Their favorable biological properties—including biocompatibility, low immunogenicity, and inherent targeting capabilities—render them ideal vectors for drug delivery. These attributes are particularly valuable in the development of innovative strategies aimed at overcoming chemoresistance in cancer therapy [158].

### 5.1 Engineering Exosomes as Drug Carriers

Genetic modification of exosomes is an effective way to confer new functions. This is achieved by fusing targeting ligands or peptides to exosomal transmembrane proteins, which are expressed in donor cells via plasmid transfection. The resulting exosomes display these ligands on their surface [159]. To date, several studies have investigated the use of exosomes as drug delivery vehicles. Tians’s comparative study showed that engineered exosomes displaying internalizing RGD peptide (iRGD) can effectively deliver DOX to αv integrin–positive breast cancer cells, resulting in significant tumor inhibition with minimal systemic toxicity [160]. Additional studies on breast cancer developed Tyr-Ile-Gly-Ser-Arg peptide (YIGSR) functionalized hybrid exosomes by combining exosomes from macrophages with lipid-polymeric nanoparticles to target laminin receptors overexpressed in breast cancer cells. These exosomes effectively delivered dasatinib, significantly enhancing drug uptake, inducing oxidative stress, and increasing apoptosis in tumor cells compared to non-targeted forms. *In vivo*, the system showed a 20-fold increase in drug bioavailability and a nearly 7-fold reduction in tumor size, demonstrating strong potential for precision breast cancer therapy [161]. The study by Wan presented a novel drug delivery system using extracellular vesicles (EVs) conjugated with aptamers to specifically target cancer cells. The study demonstrated that these targeted EVs effectively deliver anticancer drugs to breast cancer cells, enhancing therapeutic efficacy while minimising side effects [162]. Another researcher focused on PC and highlights engineered exosomes as highly promising drug carriers for this aggressive and hard-to-treat malignancy. Exosomes have been modified in several ways to enhance their drug delivery potential: by adding chondroitin sulfate for targeting CD44-positive tumor cells, using metabolic glycoengineering for precise delivery via click chemistry, and reassembling them with photosensitizers like chlorin e6 for imaging-guided photodynamic therapy. These strategies improve targeting, drug accumulation, and therapeutic precision in hard-to-treat pancreatic tumors [163]. Recently, investigators have examined advanced nanoparticle-based drug delivery systems (NDDS) for targeted CRC treatment. Researchers developed and characterised three distinct formulations: Bevacizumab-loaded chitosan nanoparticles (BEV-CHI-NP), polymeric micelles (BEV-PM), and BEV-conjugated exosomes enriched with AS1411 nucleolin-targeting DNA aptamer (AS1411) and N1-methyladenosine (AP-BEV + M1A-EXO). Among these, the AP-BEV + M1A-EXO formulation demonstrated superior efficacy, achieving a 65.4% reduction in tumor volume and significant decreases in tumor biomarkers carcinoembryonic antigen (CEA) and carbohydrate antigen 19-9 (CA 19-9), highlighting its potential as a precise and effective therapeutic strategy for CRC [164].

Alharbi et al. explored the use of nanotechnology to enhance the therapeutic effects of mangiferin and curcumin against OC. Researchers developed exosomal and liposomal nano-carriers to deliver these phytochemicals, aiming to improve their bioavailability and target the overactive PI3K/Akt/mTOR signaling pathway, which is central to OC progression. Computational analyses, including molecular docking and 100-nanosecond molecular dynamics simulations, demonstrated strong binding affinities of mangiferin and curcumin to key proteins in the pathway, suggesting that this nanotechnological approach could be a promising strategy in precision therapy for OC [165]. Interesting findings were made by Luo et al., who investigated the therapeutic potential of exosomes derived from expanded natural killer (eNK) cells in treating OC. These eNK-derived exosomes (eNK-EXO) were found to express typical NK cell markers and cytotoxic substances, demonstrating inherent anti-tumour activity against OC cells. Furthermore, when loaded with CIS, eNK-EXO enhanced the drug’s cytotoxic effects on resistant OC cells and reactivated suppressed NK cells within the TME, suggesting a dual mechanism of direct tumor inhibition and immune system modulation [166]. Pourmasoumi et al. explored the development of a targeted drug delivery system using exosomes conjugated with folic acid to deliver temozolomide (TMZ) and quercetin (QT) to glioblastoma (GBM) cells. Folic acid was utilized to enhance the targeting of exosomes to GBM cells that overexpress folate receptors. The co-delivery of TMZ and QT via these engineered exosomes resulted in increased cytotoxicity and apoptosis in GBM cells compared to treatments with either drug alone, indicating a synergistic effect and potential for improved therapeutic outcomes in GBM treatment [167]. Another study on GBM presents the development of hybrid nanocarriers combining exosomes and liposomes for targeted GBM treatment. These nanocarriers are labelled with a lipophilic NIR-II cyanine dye (NIR-C_12_) and functionalized with cyclic RGD peptides to enhance tumour targeting. The resulting NIR-C_12_-EL exhibits excellent colloidal stability and a high photothermal conversion efficiency of 62.28%, enabling effective NIR-II fluorescence imaging and photothermal therapy, which collectively contribute to prolonged survival in glioblastoma-bearing mice [168]. In a separate study, other researchers employed a similar exosome-based delivery strategy for Withaferin A. Withaferin A, a natural compound with anti-angiogenic properties, has been delivered using exosomes to enhance its therapeutic efficacy. Both intraperitoneal and oral administration of exosome-loaded Withaferin A showed a much stronger anti-tumour effect compared to free drugs in a human lung cancer xenograft mouse model [169].

### 5.2 Efflux Pump Inhibitors and Delivery of Chelating Agents

One of the major mechanisms behind chemoresistance is the upregulation of drug efflux transporters such as P-glycoprotein (P-gp/ABCB1) and multidrug resistance-associated proteins (MRPs). These ABC transporters actively remove cytotoxic drugs from the intracellular space, reducing their effectiveness. Engineered exosomes have shown promise in overcoming this mechanism by delivering inhibitors of these pumps directly to tumor cells, thereby enhancing intracellular drug retention and restoring chemosensitivity. In the literature, “pump inhibitors” delivered in exosomes are usually RNA molecules (miRNAs or siRNAs) targeting key regulatory genes associated with ABC-transport function [26,159,170].

Furthermore, exosomes have been explored as carriers for metal-chelating agents. Metal ions such as iron and copper are dysregulated, which has been implicated in both tumorigenesis and neurodegenerative disorders. Targeted delivery of chelators via exosomes may limit systemic toxicity and increase drug accumulation in specific tissues. This approach may increase drug accumulation in specific tissues and limit systemic toxicity [171–173].

Bellingham et al. discussed the potential of extracellular vesicles, particularly exosomes, as innovative carriers for targeted delivery of metal-chelating drugs to address disrupted metal ion homeostasis in neurodegenerative disorders. The authors emphasised that utilising exosomes for chelator delivery could enhance therapeutic specificity and efficiency while minimizing systemic toxicity. Furthermore, this strategy offers promising implications for precision medicine in treating conditions characterized by pathological metal accumulation, such as Alzheimer’s and Parkinson’s diseases [171]. Tao and Gao reviewed the potential of exosomes for diagnosing and treating neurodegenerative diseases. They emphasised exosomes’ ability to cross the blood-brain barrier, enabling targeted delivery of therapeutic agents, including metal-chelating drugs. Their analysis suggested that this approach could significantly improve the specificity and effectiveness of treatments for neurodegenerative disorders [172]. Nouri et al. further supported findings by Tao and Gao 2024 and Bellingham et al., highlighting exosomes as effective vehicles capable of crossing the blood-brain barrier to deliver targeted therapies, including metal-chelating drugs, for neurodegenerative diseases [173].

In the context of cancer, exosomes have been explored as drug delivery systems capable of transporting chemotherapeutics like paclitaxel and DOX directly to tumor cells, reducing systemic toxicity. Although their use for delivering metal-chelating agents in oncology is still limited, their potential in targeting tumors with metal ion dysregulation is significant [174].

### 5.3 Genetic Cargo Delivery: mRNA and miRNA

Exosomes can also be modified in order to deliver genetic material capable of modulating cellular pathways involved in drug resistance. To re-sensitise tumor cells to chemotherapeutic agents, miRNAs that downregulate resistance-associated genes or mRNAs encoding pro-apoptotic proteins can be packaged into exosomes [158,175].

Su et al. demonstrated that increasing the expression of miR-135b in drug-resistant non-small cell lung cancer (NSCLC) cells enhanced their sensitivity to CIS treatment by directly downregulating Frizzled-1 (FZD1) [176]. Zeng et al. investigated the role of exosomal miR-151a in glioblastoma multiforme (GBM). They found that miR-151a was downregulated in temozolomide (TMZ)-resistant GBM cells. Restoring miR-151a expression sensitised these cells to TMZ by inhibiting XRCC4-mediated DNA repair. Furthermore, exosomes from TMZ-resistant cells lacking miR-151a could transfer this resistance to sensitive cells. However, supplementing exosomal miR-151a reversed this effect, suggesting that exosomal miR-151a could be a therapeutic target to overcome chemoresistance in GBM [177]. Lou et al. explored the use of exosomes derived from adipose tissue-derived mesenchymal stem cells (AMSCs) modified to overexpress miR-122. They demonstrated that these exosomes could effectively deliver miR-122 to hepatocellular carcinoma (HCC) cells, leading to increased sensitivity to chemotherapeutic agents like sorafenib. *In vivo* experiments further showed that intra-tumour injection of these exosomes enhanced the antitumor efficacy of sorafenib, indicating that AMSC-derived exosomes carrying miR-122 could be a novel strategy to improve HCC chemosensitivity [178].

Exosomal delivery of therapeutic mRNAs represents a promising strategy to counteract resistance mechanisms in cancer.

### 5.4 Combining Exosome-Based Therapies with Conventional Drugs

Integrating exosome-based therapies with traditional anticancer agents aims to exploit the natural targeting abilities of exosomes to enhance drug delivery and efficacy. This combination can potentially:

- Enhance Drug Delivery: Exosomes can be engineered to carry chemotherapeutic drugs directly to tumor cells, improving drug accumulation at the tumor site and reducing systemic toxicity [179].
- Reverse Drug Resistance: By delivering specific molecules that inhibit resistance pathways, exosomes can sensitize tumor cells to conventional therapies [180].
- Modulate Immune Responses: Exosome-based treatments combined with immunotherapies can enhance anti-tumor immunity, potentially overcoming resistance mechanisms [181].

### 5.5 Examples of Combination Therapy Applications

Numerous studies have demonstrated the advantages of combining therapeutic agents, particularly in the management of complex or multifactorial diseases. In preclinical studies, bone marrow-derived mesenchymal stem cell (MSC) exosomes loaded with PAC successfully targeted breast cancer cells, increasing drug efficacy while reducing systemic toxicity [169]. Building on this concept, further research has demonstrated that exosomes can serve as efficient nanocarriers for PTX, significantly inhibiting tumor growth and metastasis. In particular, PTX-loaded exosomes have been shown to enhance drug accumulation at tumour sites while minimizing off-target effects [182,183]. In addition to PTX delivery, exosomes have also been employed as carriers for DOX, particularly in the context of drug-resistant cancers. Researchers have explored loading DOX into exosomes to target drug-resistant cancer cells. The exosome-mediated delivery system enhanced the cytotoxicity of DOX against resistant breast cancer cells, suggesting a potential strategy to overcome drug resistance [184]. Expanding on this strategy, another investigation focused on the co-delivery of DOX and miRNA-159 using targeted exosomes in triple-negative breast cancer (TNBC) models. This combined delivery system was shown to silence the T-cell factor 7 (TCF-7) gene effectively, resulting in enhanced anticancer activity without inducing significant toxicity. The study demonstrated a synergistic interaction between DOX and miR-159, offering a promising approach to overcoming therapeutic resistance in TNBC [185]. Further innovations have included engineering exosomes with dual-targeting ligands to optimize delivery efficiency. In a 2023 study, researchers developed exosomes functionalized with both iRGD and tLyp1 peptides, designed to improve specificity and uptake by breast cancer cells. These modified exosomes significantly enhanced DOX accumulation in both MCF-7 and MDA-MB-231 cell lines, leading to increased cytotoxic effects. This dual-targeting strategy offers a promising avenue for addressing drug resistance through more precise tumor targeting [186]. Another study showed that boron clusters (B_12_Br_12^2−^_) enhanced DOX loading into milk-derived exosomes, creating exosome–dodecaborate (EDB) particles that overcame DOX resistance in breast cancer. EDB improved intracellular DOX delivery, inhibited P-glycoprotein-mediated efflux, and restored drug sensitivity, effectively suppressing tumor growth in resistant models [187].

Research conducted by Zhang et al. on OC demonstrated that CIS-loaded exosomes from umbilical cord blood M1 macrophages significantly boost cytotoxicity in OC cells, offering a promising strategy to overcome drug resistance [188]. Other studies by [189] have shown that exosomes from omental adipocytes influence epithelial ovarian cancer (EOC) progression and resistance to paclitaxel chemotherapy. The researchers found that these exosomes induce epithelial-to-mesenchymal transition (EMT) in EOC cells, enhancing their invasive capabilities. Additionally, the exosomes reduced the efficacy of PTX by promoting cancer cell proliferation and survival. MiRNA sequencing identified several miRNAs—miR-21, let-7b, miR-16, and miR-92a—as abundant in these exosomes, contributing to the observed effects [189]. Following this, X. Pan et al. examined the role of exosomal miR-4516 in promoting CIS resistance in OC. Researchers found that ovarian cancer stem cells (OCSCs) secrete exosomes enriched with miR-4516, which are taken up by CIS-resistant ovarian cancer cells (SKOV3/DDP). This uptake leads to the downregulation of the tumor suppressor gene GAS7, thereby enhancing the cells’ resistance to CIS. Overexpression of GAS7 was shown to reverse this resistance, both *in vitro* and *in vivo*, suggesting that targeting the miR-4516/GAS7 axis could be a potential therapeutic strategy to overcome CIS resistance in OC [190]. In a separate study, [191] explored that exosomal lncRNA PLADE from ascites enhances CIS sensitivity in high-grade serous OC by promoting R-loop formation. PLADE downregulates HNRNPD via VHL-mediated degradation, leading to DNA damage and increased apoptosis, and is notably reduced in CIS-resistant patients. Another experiment that explored the role of exosomes in reducing drug resistance was conducted by Zhao et al. The study showed that exosomes loaded with tetramethylpyrazine (EXO-TMP) reversed PTX resistance in A2780T OC cells by downregulating P-gp, ABCC1, and glutathione S-transferase Pi 1 (GSTP1) GSTP1, leading to increased apoptosis and improved drug sensitivity [192].

Another emerging area of interest involves the role of exosomes in overcoming resistance mechanisms in CML. The study by Du et al. showed that modified dendritic cell-derived exosomes (Dex) overcame drug resistance in CML, including T315I mutation, by activating NK and T cells via the NKG2D/NKG2D-L pathway, leading to effective leukemia cell killing and tumor suppression in mice [193]. Bellavia et al. engineered exosomes to express the interleukin-3 (IL-3) receptor ligand, allowing them to specifically target CML cells. These exosomes were loaded with either imatinib or BCR-ABL siRNA. The study demonstrated that these targeted exosomes effectively inhibited the growth of CML cells both *in vitro* and *in vivo*, suggesting a potential strategy to overcome drug resistance [194]. Javidi-Sharifi et al. found that fibroblast growth factor 2 (FGF2) from bone marrow stromal cells is secreted in exosomes, which are then taken up by leukemia cells, protecting them from tyrosine kinase inhibitors (TKIs). Inhibiting the fibroblast growth factor 2 and fibroblast growth factor receptor 1 (FGF2-FGFR1) signaling pathway reduced exosome secretion and sensitized leukemia cells to TKIs, indicating a potential therapeutic approach to counteract drug resistance [195].

Pancreatic cancer (PC) is an additional example of a malignancy characterized by poor prognosis and significant resistance to therapy. The study by Zhou et al. showed that bone marrow mesenchymal stem cell-derived exosomes loaded with PTX and gemcitabine monophosphate effectively penetrated pancreatic tumors, enhanced drug delivery, and inhibited tumor growth, offering a targeted strategy with reduced toxicity [196]. In pancreatic ductal adenocarcinoma (PDAC), exosomal miR-155 and circBIRC6 have been shown to promote gemcitabine and oxaliplatin resistance, respectively, by enhancing anti-apoptotic signaling and DNA repair pathways [66].

Liver cancer, particularly hepatocellular carcinoma, also poses significant therapeutic challenges. According to findings by Hwang et al., they showed that exosomal miR-6126 restores sorafenib sensitivity in resistant hepatocellular carcinoma by targeting CD44 and HK2, making it a promising therapeutic target to overcome drug resistance [197]. Another study demonstrated that engineered exosomes delivering a ferroptosis-inducing agent enhanced the efficacy of sorafenib in hepatocellular carcinoma. By combining exosome-based targeted delivery with a conventional drug, the approach helped overcome sorafenib resistance and boosted cancer cell death via ferroptosis [198]. The study by Li et al. showed that exosomes from bone marrow mesenchymal stem cells (BM-MSCs) modified with siRNA against GRP78 effectively delivered the siRNA to hepatocellular carcinoma cells, downregulated GRP78, and restored sorafenib sensitivity. Combined treatment significantly inhibited tumor growth and metastasis in resistant HCC models [199].

In the context of central nervous system malignancies, glioblastoma multiforme (GBM) stands out due to its poor prognosis and limited therapeutic success. The study by Han et al. introduced an innovative drug delivery system designed to traverse the blood-brain barrier (BBB) and enhance the efficacy of chemotherapeutic agents. This system utilizes engineered exosome membranes to camouflage thermoresponsive nanoparticles, enabling targeted delivery and controlled release of drugs within the brain. Although the study does not directly address drug resistance, the exosome-camouflaged, thermoresponsive delivery system enhances drug accumulation in brain tumors and may help overcome resistance by improving targeted penetration and controlled release across the blood-brain barrier [200]. Another study by Pourmasoumi et al. presents a novel approach to glioblastoma treatment by co-delivering temozolomide (TMZ) and quercetin (Qct) using folic acid (FA)-conjugated exosomes [167]. This strategy aims to overcome TMZ resistance and enhance drug delivery across the BBB. In the study by [201], glioblastoma cells were found to secrete exosomes containing the long noncoding RNA lnc-TALC. These exosomes are taken up by microglia, leading to their polarization into the M2 phenotype and subsequent production of complement component C5. The C5/C5a signaling pathway enhances DNA repair mechanisms in glioblastoma cells, contributing to resistance against TMZ chemotherapy. To counteract this resistance, the researchers employed a C5a receptor (C5aR1) antagonist, which effectively disrupted the exosome-mediated communication between glioblastoma cells and microglia. This intervention restored the sensitivity of glioblastoma cells to temozolomide, highlighting a potential therapeutic strategy to overcome chemoresistance in GBM [201]. Other researchers have contributed to this topic by examining the role of pacritinib, a STAT3 inhibitor, in overcoming TMZ resistance in glioblastoma. They found that M2-polarized glioblastoma-associated macrophages (GAMs) secrete exosomes enriched with miR-21, which contribute to TMZ resistance in glioblastoma cells. Treatment with pacritinib downregulated STAT3 activity in these macrophages, leading to a reduction in miR-21-enriched exosome secretion. This, in turn, sensitized glioblastoma cells to TMZ, suggesting that targeting the STAT3 pathway in GAMs can mitigate exosome-mediated chemoresistance [202]. In the study by Munoz et al., the authors identified the Sonic Hedgehog (SHH) signaling pathway as a key contributor to TMZ resistance in GBM cells. They discovered that miR-9 suppresses PTCH1, a negative regulator of SHH signaling, leading to ligand-independent activation of the SHH pathway and upregulation of the multidrug resistance gene MDR1. To counteract this, the researchers utilized bone marrow-derived mesenchymal stem cells (MSCs) engineered to deliver anti-miR-9 via exosomes and gap junctions. This approach successfully restored PTCH1 expression, inhibited SHH signaling, and sensitized GBM cells to TMZ, suggesting a potential therapeutic strategy to overcome chemoresistance [203].

In addition to the cancers discussed above, melanoma also presents distinct clinical and molecular challenges. Combining exosome-targeted strategies with conventional chemotherapy may improve treatment outcomes in drug-resistant cancers, as supported by recent studies. Kulkarni et al. demonstrated that growth hormone (GH) promotes drug resistance and migration in melanoma by enhancing the release of exosomes enriched with ABCC1, ABCB1, and other resistance-related proteins. Importantly, inhibition GH signaling with the receptor antagonist pegvisomant reduced the exosomal transfer of these factors and restored drug sensitivity. This work highlights how interfering with exosome-mediated communication can enhance the efficacy of classical anticancer therapies [204]. In the study by Feng et al., researchers developed an innovative cancer treatment strategy by combining exosome-based delivery with conventional photodynamic therapy to overcome drug resistance in melanoma. They engineered coordination-assembled Iridium(III) photosensitizers camouflaged with exosome membranes, enhancing tumor targeting and biocompatibility. Upon light activation, these nanoparticles induced multiple forms of cell death—apoptosis, autophagy, and ferroptosis—effectively eradicating melanoma tumors and inhibiting metastasis in mouse models. This approach exemplifies how integrating exosome-based delivery systems with traditional therapies can improve outcomes in drug-resistant cancers [205].

Nittayaboon et al. conducted a proteomic analysis of exosomes derived from butyrate-resistant CRC cells and identified proteins potentially involved in resistance to multiple anticancer drugs. These exosomal proteins may facilitate intercellular transfer of resistance traits, contributing to reduced treatment efficacy. Targeting such exosome-mediated mechanisms could help restore drug sensitivity and enhance the effectiveness of standard therapies in resistant CRC [206].

Lung cancer, one of the leading causes of cancer-related mortality worldwide, also demonstrates considerable treatment resistance. In the study by Saeed et al., researchers explored the use of camel milk-derived exosomes as nanocarriers for delivering curcumin (CUR) to lung cancer cells. They successfully isolated exosomes from camel milk and loaded them with CUR, achieving a 20% loading efficiency. *In vitro* experiments demonstrated that the exosome-encapsulated curcumin (ExoCUR) exhibited significantly enhanced cytotoxic effects against both drug-sensitive (A549) and taxol-resistant (A549TR) lung cancer cell lines compared to free curcumin. Molecular docking and dynamics simulations indicated that CUR has a strong binding affinity for the epidermal growth factor receptor (EGFR), comparable to the established drug gefitinib. Furthermore, CUR effectively downregulated EGFR and STAT3 expression in lung cancer cells, suggesting its potential to disrupt key signaling pathways involved in tumor progression [207]. These findings highlight the potential of camel milk-derived exosomes as effective and biocompatible delivery systems for curcumin, offering a promising strategy to overcome drug resistance in lung cancer therapy.

### 5.6 Exosome-Based Therapy—How to Study It?

Currently, the most optimal method for studying exosomes and testing exosome-based therapy seems to be xenograft *in vivo* models. Xenotransplants are widely used for the analysis of cancer cells’ tumorigenicity, tumor histology, and for testing of tumor response to novel therapies [208]. Many studies have already been undertaken using this model, due easy to label exosomes. For instance, the application of C-X-C chemokine receptor 4 (CXCR4)/TNF-related apoptosis-inducing ligand (TRAIL)-enriched exosomes in improving the efficacy of brain disease chemotherapy was tested [209]. NK cell-derived exosomes were studied in the primary liver cancer, hepatocellular carcinoma, using the orthotopic and subcutaneous tumor model also. Both are xenograft models but obtained by different techniques [210]. The other scientist analysed hypoxic exosomes on a mouse glioblastoma multiforme xenograft model [211].

Plasma of acute myeloid leukaemia (AML) patients is significantly enriched in exosomes. Chang-Sook Hong et al. showed that fully engrafted AML PDX (patient-derived xenograft) mice produced exosomes with properties similar to those of exosomes isolated from AML patients’ plasma [212,213]. This is particularly important because we know that most research models are not perfect and do not fully reflect the conditions prevailing in the patient’s body. Therefore, we believe that research using exosome-based therapy may yield the most and fastest positive results in AML research, rather than in other cancers.

However, when using exosome-based therapy, we must always remember the existing limitations. In the article, we mentioned several times the high heterogeneity of exosomes, which makes standardisation and schematic analysis difficult. The heterogeneity is often present within EVs isolated from a single cell type [214]. The other problem lies in achieving stable, large-scale production of exosomes. It is important to take into account established dosage plans for exosome treatment because of exosomes’ short half-life, which leads to short circulation time *in vivo* [215]. Key challenges include identifying the administration route. The costs of such therapy are still very high, making it difficult to disseminate on a large scale [158].

Nevertheless, exosomes, due to their low immunogenicity and capacity to pass through small pores, hold great promise in cancer therapy [181].

## 6 Conclusions

Exosomes are key in developing cancer resistance to cytostatics, acting as active mediators of intercellular communication within the tumor microenvironment. Through the transfer of transport proteins (such as P-gp, BCRP), immunosuppressive ligands (e.g., PD-L1, TGF-β, PGE2) and non-coding RNA molecules (miRNA, circRNA), exosomes promote the transfer of the resistant phenotype between cancer cells and between cancer cells and the surrounding microenvironment. These observations confirm their fundamental role in the modification of signaling pathways, regulation of apoptosis, angiogenesis and in shaping the immunosuppressive TME, which results in reduced efficacy of anticancer therapy.

**Figure 2:**
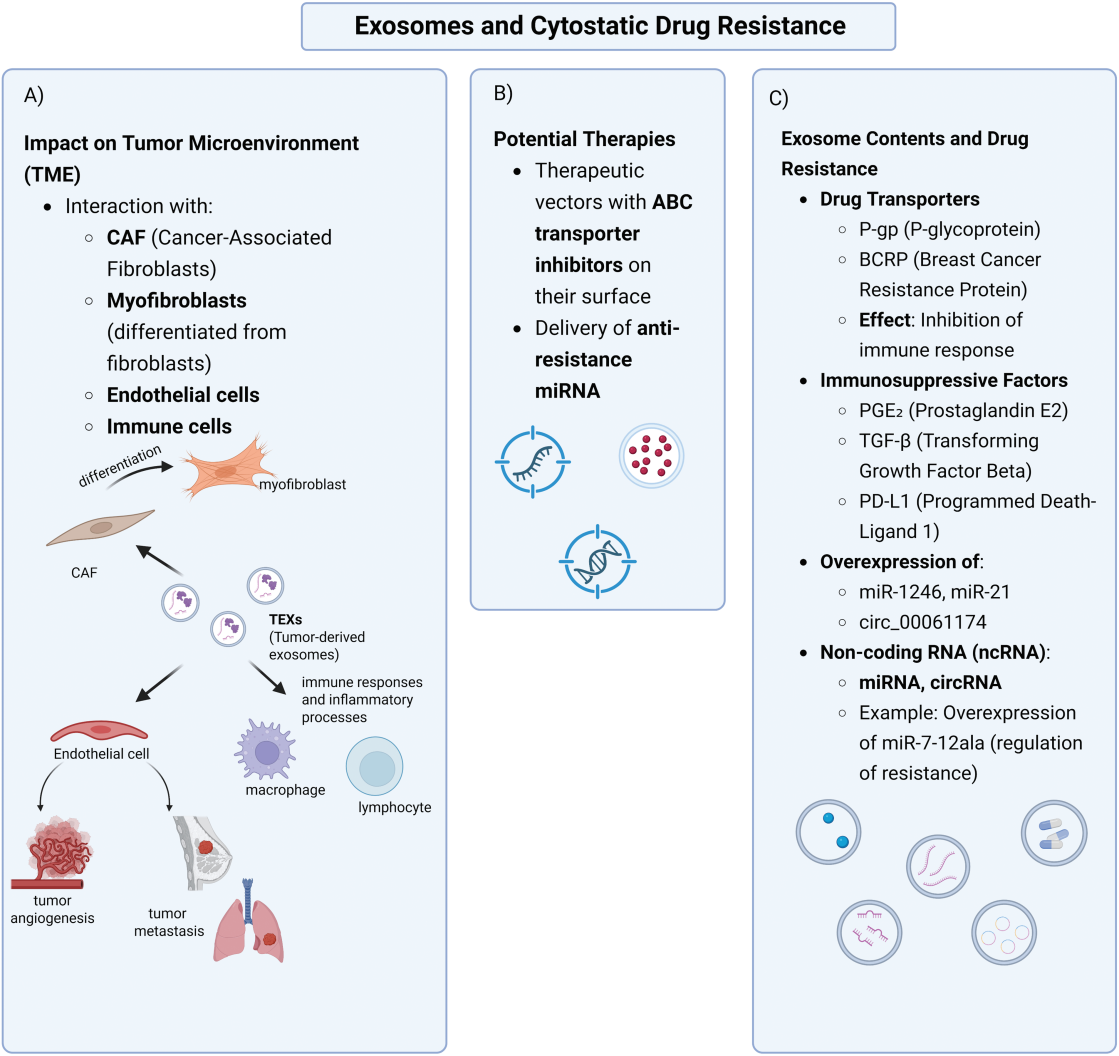
Exosomes and cytostatic drug resistance. (**A**) Tumour-derived exosomes (TEXs) interact with components of the tumour microenvironment (TME), including cancer-associated fibroblasts (CAFs), myofibroblasts, endothelial, and immune cells, contributing to TME remodelling, angiogenesis, metastasis, and immunomodulation. (**B**) Potential therapies involve exosome-based vectors delivering ABC transporter inhibitors or anti-resistance miRNAs. (**C**) Exosomes carry drug transporters (e.g., P-gp, BCRP), immunosuppressive factors (PGE_2_, TGF-β, PD-L1), and non-coding RNAs (miRNAs, circRNAs), which mediate drug resistance and immune escape. Overexpression of miR-1246, miR-21, and circ_0006174 is particularly implicated in multidrug resistance (Created in BioRender, accessed on 10 October 2024)

Due to their presence in various body fluids and the ability to selectively transfer bioactive molecules, exosomes have great potential as non-invasive biomarkers used in diagnostics, monitoring treatment efficacy and prognosis of the course of cancer. At the same time, their participation in shaping therapeutic resistance makes them an attractive target for therapeutic interventions—both through blocking their secretion or inhibiting the transport of resistance, and through engineering exosomes as carriers of anticancer drugs. However, the clinical application of these strategies requires further deepening of knowledge about the biology of exosomes and their interactions within the tumor microenvironment (Fig. 2).

Despite the dynamic development of this field, many mechanisms of exosome action remain incompletely understood. Furthermore, we have to remember many limitations associated with exosome research itself. Currently, there’s no single, standardised protocol for exosome isolation due to their biodiversity [216]. Despite extensive efforts, *in vitro* analysis of isolated vesicles may still not fully reflect the conditions found in living organisms. Therefore, it is necessary to conduct further, targeted experimental and translational studies that will allow for a better understanding of their role in the process of resistance to treatment and will enable the development of effective therapeutic strategies based on their modification or modulation. Integrating knowledge from molecular biology, tumor immunology and nanomedicine may contribute to a future breakthrough in the treatment of chemotherapy-resistant tumors. At the same time, we must remember about nutritional aspects and preventive oncology, which can complement each other. And therapeutic interventions should be individualised [217].

## Data Availability

Not applicable.
